# The Diagnostic Accuracy of Ex Vivo Confocal Laser Scanning Microscopy for Squamous Cell Carcinoma: A Systematic Review and Meta-Analysis

**DOI:** 10.3390/diagnostics16101539

**Published:** 2026-05-19

**Authors:** Luis Messner, Sarah Lukacs, Michael J. Flaig, Daniela Hartmann, Benjamin Kendziora

**Affiliations:** 1 Department of Dermatology and Allergy, University Hospital, LMU, 80337 Munich, Germany; 2 Department of Dermatology, Thalkirchner Street Hospital, Munich Municipial Hospital Group, 80337 Munich, Germany

**Keywords:** carcinoma, squamous cell, confocal laser scanning microscopy, diagnostic accuracy, dermatologic surgical procedures, Mohs surgery, meta-analysis

## Abstract

**Background/Objectives:** Complete excision of squamous cell carcinoma (SCC) while preserving healthy tissue relies on accurate diagnosis and assessment of tumor margins. Ex vivo confocal laser scanning microscopy (EVCM) allows rapid, high-resolution visualization of freshly excised tissue. This study was conducted to comprehensively assess the diagnostic accuracy of EVCM for SCC diagnosis in tissue specimens and margin assessment in margin-controlled (micrographic) surgery using conventional histopathology as the reference standard. **Methods:** A systematic literature search of MEDLINE and Embase was conducted on 1 January 2026, in accordance with PRISMA guidelines. Pooled sensitivity and specificity were estimated using bivariate random-effects models. The QUADAS-2 and GRADE frameworks were applied to assess risk of bias and certainty of evidence. **Results:** Six studies comprising a total of 288 specimens were included. For SCC diagnosis in tissue specimens, the pooled sensitivity was 85.1% (95% confidence interval [CI]: 71.6–92.8) and the pooled specificity was 95.5% (95% CI: 90.9–97.8), with low between-study heterogeneity and moderate certainty of evidence. For margin assessment, pooled sensitivity and specificity were 89.9% (95% CI: 51.6–98.7) and 96.1% (95% CI: 85.8–99.0), respectively, with low heterogeneity but also low certainty of evidence owing mainly to the limited number of included studies and specimens. **Conclusions:** EVCM demonstrates moderate sensitivity and high specificity for the diagnosis of SCC in tissue specimens and may be used selectively as an adjunct to conventional histology, for example as a rapid confirmatory diagnostic tool capitalizing on its high specificity. Current evidence for margin assessment, although promising, remains limited.

## 1. Introduction

Cutaneous squamous cell carcinoma (SCC) is the second most common skin cancer worldwide, with an increasing incidence and a substantial risk of metastasis or recurrence, particularly in the presence of high-risk features, including tumor diameter > 20 mm; localization on the lip, ear, or temple; thickness > 6 mm; or invasion beyond subcutaneous fat, poor differentiation, desmoplasia, perineural invasion, bone erosion, immunosuppression, or positive surgical margins [1,2,3,4]. Accurate clinical history, histological diagnosis, and precise assessment of tumor margins are therefore critical for effective patient management. Although conventional histopathological examination of excised tissue is reliable and remains the diagnostic gold standard, it is resource-intensive, time-consuming, and can delay decision-making [5,6].

Ex vivo confocal laser microscopy (EVCM) has emerged as an innovative imaging modality that enables rapid, high-resolution visualization of skin structures at both cellular and subcellular levels within minutes. By replicating the appearance of conventional hematoxylin–eosin (H&E) staining, advanced digital staining algorithms have the potential to accelerate diagnostic processes in a wide range of skin diseases, support margin assessment, and enhance surgical workflows [7,8]. While EVCM has demonstrated the ability to identify characteristic features of SCC and support its diagnosis, its overall diagnostic accuracy remains uncertain, largely due to the nature of the predominantly small, single-center studies and heterogeneous study protocols [5,9].

To evaluate the diagnostic performance of EVCM, we conducted a systematic review and meta-analysis summarizing and analyzing available data from the literature on its accuracy both for the diagnosis of SCC in tissue specimens and for the margin assessment in margin-controlled (micrographic) surgery, using conventional histopathology as the reference standard.

## 2. Materials and Methods

### 2.1. Protocol Registration and Reporting Standards

This systematic review and meta-analysis was conducted according to the Preferred Reporting Items for Systematic Reviews and Meta-Analyses (PRISMA) of Diagnostic Test Accuracy Studies guidelines [10]. The study protocol was developed prior to initiation of the database searches and registered with the International Prospective Register of Systematic Reviews (PROSPERO; ID: CRD420251262311) during the database search process and before data analysis commenced.

### 2.2. Search Strategy and Study Selection

A systematic literature search was carried out in MEDLINE and Embase (via Ovid) on 1 January 2026, to identify studies evaluating the diagnostic accuracy of EVCM in the diagnosis and/or margin assessment of SCC. The complete search strategy is provided in Supplementary Table S1.

Studies were eligible for inclusion if they: investigated the diagnostic accuracy of EVCM for the diagnosis of SCC in tissue specimens and/or margin assessment in micrographic surgery; reported a 2 × 2 contingency table or provided sufficient information to reconstruct one; and used formalin-fixed paraffin-embedded or cryoconserved histology as the reference standard.

Studies were excluded if the EVCM evaluation of SCCs was performed exclusively by artificial intelligence (AI) or automated image analysis without subsequent human expert validation.

No restrictions regarding publication year were applied. Conference abstracts, case reports, review articles, and opinions were excluded. Furthermore, studies including fewer than 10 cases of SCC were not eligible for inclusion.

The reference lists of all included studies and relevant reviews in the field were manually screened to identify further eligible publications, which might have been missed in the database search (reference mining).

### 2.3. Data Extraction

Data extraction was performed independently by two reviewers (L.M. and S.L.) using a standardized, predefined extraction template. The data extracted included study design, characteristics of patients and lesions, specimen preparation techniques (including punch biopsy, shave biopsy, bread-loaf excision, and micrographic surgery via the Mohs technique [11,12], margin-strip method = “Tuebinger Torte” [13], muffin technique [13], or other), the type of EVCM device used, EVCM image acquisition time, the used reference standard (either formalin-fixed paraffin-embedded or cryoconserved histology), as well as diagnostic accuracy outcomes (true positives, false positives, false negatives, and true negatives).

All extracted information was subsequently compared between reviewers to ensure accuracy and consistency. When discrepancies or unclear data were identified, the authors of the original studies were contacted for further clarification. Any disagreements were resolved by discussion, and if necessary, a third reviewer (B.K.) was consulted to reach a final decision.

### 2.4. Data Synthesis and Statistical Analysis

All statistical analyses were performed using R software (version 4.5.2; R Foundation for Statistical Computing, Vienna, Austria). The analyses were conducted by one author (B.K.), who holds a Master’s degree in Biostatistics, using the meta [14] and mada R packages [15].

In diagnostic test accuracy studies, sensitivity and specificity are typically correlated, as both depend on the diagnostic threshold applied. For this reason, Rutter and Gatsonis [16] and Reitsma et al. [17] proposed bivariate random-effects models for meta-analysis of diagnostic accuracy data, which jointly model sensitivity and specificity while accounting for their correlation. We used bivariate random-effects models following the Reitsma et al. framework [17] to pool sensitivity and specificity estimates of EVCM, using conventional histology (either formalin-fixed paraffin-embedded or cryoconserved histology, depending on its use in the individual included studies) as the reference standard. Sensitivities and specificities from studies evaluating the diagnostic accuracy of EVCM for the diagnosis of SCC in tissue specimens were pooled separately from those assessing its accuracy for margin control in micrographic surgery.

Given the technological evolution of EVCM devices, a robustness/sensitivity analysis was performed to compare earlier- and newer-generation systems regarding their diagnostic accuracy for the detection of SCC. A corresponding meta-regression was applied to assess potential differences in accuracy between device generations. For margin assessment, this sensitivity analysis could not be conducted due to insufficient data.

A predefined subgroup analysis to compare the diagnostic accuracy of EVCM across micrographic surgery techniques could not be performed because of insufficient data.

A two-sided α-level of 0.05 was considered statistically significant for all analyses.

### 2.5. Certainty of Evidence Assessment

Between-study heterogeneity was evaluated using the I^2^ statistic derived from the bivariate random-effects models applied to pool sensitivity and specificity estimates for SCC diagnosis in tissue specimens and for margin assessment, respectively.

The methodological quality and applicability of all included studies were assessed using the Quality Assessment of Diagnostic Accuracy Studies (QUADAS-2) tool [18]. Two independent reviewers (L.M. and S.L.) evaluated four key domains: patient selection, index test, reference standard, and flow and timing. For each domain, the risk of bias was rated as “low,” “high,” or “unclear,” and applicability concerns were judged for the first three domains. Disagreements were resolved through discussion. When consensus could not be reached, a third reviewer (B.K.) was consulted and decisions were made by majority vote.

Potential publication bias was examined visually using funnel plots of diagnostic odds ratios for studies evaluating the accuracy of EVCM in SCC diagnosis in tissue specimens and in margin assessment during micrographic surgery. Funnel plot asymmetry was additionally assessed using the Harbord regression test [19].

Finally, the overall certainty of evidence for each pooled diagnostic accuracy outcome was determined according to the Grading of Recommendations Assessment, Development, and Evaluation (GRADE) approach for diagnostic tests [20]. Certainty ratings were reduced when there was evidence of inconsistency among study results, imprecision reflected by wide confidence intervals, inclusion of several studies with a high risk of bias, or indications of publication bias.

## 3. Results

### 3.1. Study Selection and Characteristics

**Figure 1:**
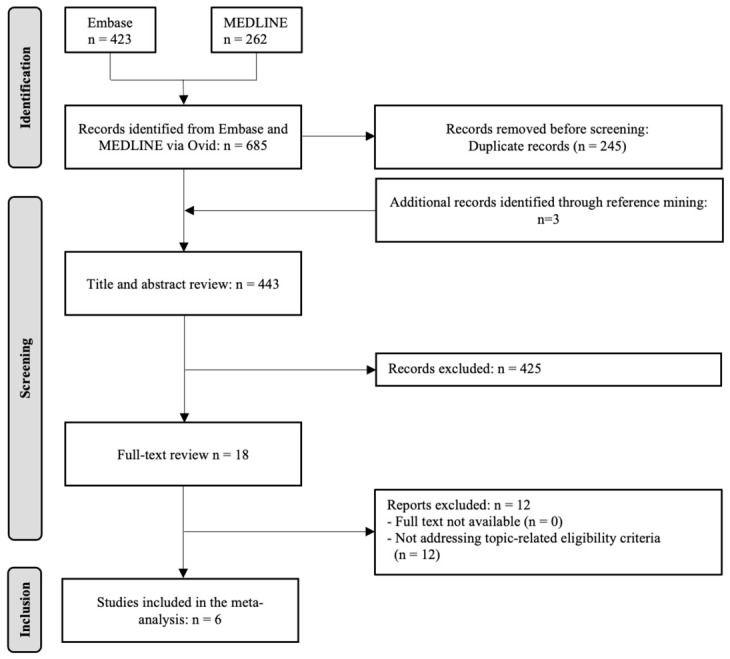
Screening of References. PRISMA flow diagram summarizing the study selection process structured into three phases: Identification, Screening and Inclusion. PRISMA = Preferred Reporting Items for Systematic Reviews and Meta-Analyses.

Our electronic database search identified a total of 685 records, which were initially screened based on titles and abstracts. Following this, 18 full-text articles were reviewed for eligibility. Of these, 6 studies met the inclusion criteria and were included in the quantitative analysis [5,7,21,22,23]. The study selection process is illustrated in the PRISMA flow diagram (Figure 1). The 6 included studies comprised a total of 288 specimens. Five studies utilized the VivaScope^®^ 2500 microscope (Lucid Inc., Rochester, NY, USA) and one study the VivaScope^®^ 1000. The main characteristics of the studies are summarized in Table 1.

### 3.2. Diagnostic Accuracy for SCC Detection

**Figure 2:**
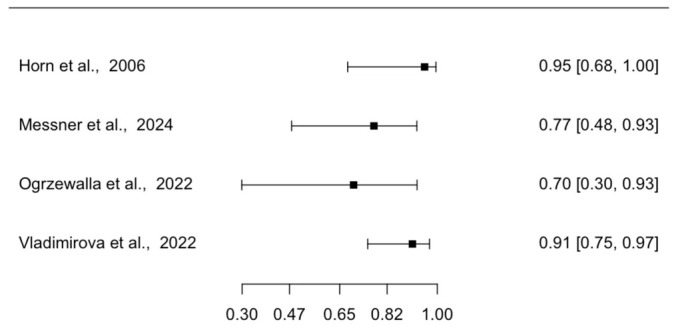
Forest Plot Illustrating the Sensitivity of EVCM for the Diagnosis of SCC in Tissue Specimens [5,7,22,23]. The plot displays individual study sensitivities with corresponding CI. EVCM = ex vivo confocal microscopy; SCC = squamous cell carcinoma; CI = confidence interval.

**Figure 3:**
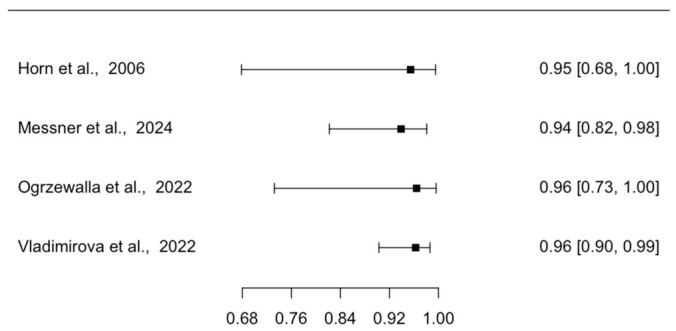
Forest Plot Illustrating the Specificity of EVCM for the Diagnosis of SCC in Tissue Specimens [5,7,22,23]. The plot displays individual study specificities with corresponding CI. EVCM = ex vivo confocal microscopy; SCC = squamous carcinoma; CI = confidence interval.

Four studies reported the diagnostic accuracy of EVCM for the diagnosis of SCC in tissue specimens with conventional histology (either formalin-fixed paraffin-embedded or cryoconserved histology) as the reference standard. Forest plots of individual study sensitivities and specificities are presented in Figure 2 and Figure 3, respectively. The pooled sensitivity was 85.1% (95% CI: 71.6–92.8), and the pooled specificity was 95.5% (95% CI: 90.9–97.8). The pooled positive likelihood ratio was 20.2 (95% CI: 9.1–39.5), and the pooled negative likelihood ratio was 0.2 (95% CI: 0.1–0.3), with an area under the summary receiver operating characteristic curve (AUC) of 0.97.

Between-study heterogeneity was low (I^2^ = 9.3%). In the QUADAS-2 domain of patient selection, the risk of bias was rated as high in two of the four included studies. In the reference standard domain, the risk of bias was rated as unclear in one study, whereas the other domains were judged to have a low risk of bias across studies. Applicability concerns regarding patient selection with respect to our research question were identified in one study (Supplementary Table S2 and Supplementary Figure S1). Visual inspection of the funnel plot of diagnostic odds ratios (Supplementary Figure S2) revealed no apparent asymmetry, which was supported by the Harbord regression test (*p* = 0.567). The pooled estimates of sensitivity and specificity were associated with relatively wide CI. On this basis, the certainty of evidence for the diagnostic accuracy for SCC diagnosis in tissue specimens was downgraded to moderate according to the GRADE framework.

### 3.3. Diagnostic Accuracy for Margin Assessment

**Figure 4:**
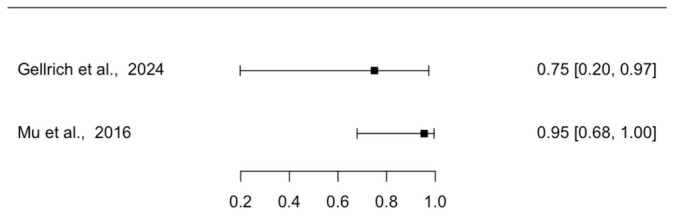
Forest Plot Illustrating the Sensitivity of EVCM for Margin Assessment in Micrographic Surgery of SCC [21,24]. The plot displays individual study sensitivities with corresponding CI. EVCM = ex vivo confocal microscopy; SCC = squamous carcinoma; CI = confidence interval.

**Figure 5:**
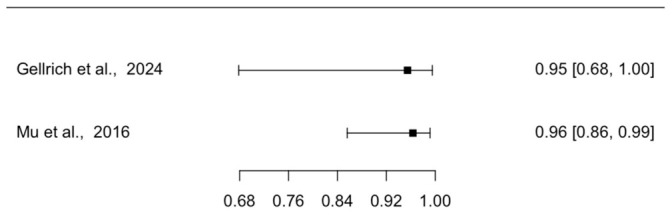
Forest Plot Illustrating the Specificity of EVCM for Margin Assessment in Micrographic Surgery of SCC [21,24]. The plot displays individual study specificities with corresponding CI, stratified by surgical technique. EVCM = ex vivo confocal microscopy; SCC = squamous carcinoma; CI = confidence interval.

Two studies reported the diagnostic accuracy of EVCM for margin assessment of SCC in micrographic surgery with conventional histology (either formalin-fixed paraffin-embedded or cryoconserved histology) as the reference standard. Forest plots of individual study sensitivities and specificities are presented in Figure 4 and Figure 5, respectively. The pooled sensitivity was 89.9% (95% CI: 51.6–98.7), and the pooled specificity was 96.1% (95% CI: 85.8–99.0). The pooled positive likelihood ratio was 28.6 (95% CI: 5.6–90.3), and the pooled negative likelihood ratio was 0.1 (95% CI: 0.0–0.5), with an AUC of 0.98.

Heterogeneity in diagnostic odds ratios was low (I^2^ = 14.1%). The study by Mu et al. raised concerns regarding risk of bias in patient selection as well as in the flow and timing domain, and also raised applicability concerns related to patient selection with respect to our research question. The study by Gellreich et al. raised applicability concerns regarding patient selection (Supplementary Table S2 and Supplementary Figure S1). Assessment of publication bias was not feasible due to the limited number of included studies. The CI around the pooled sensitivity and specificity estimates were relatively wide. Consequently, the certainty of evidence for margin assessment was downgraded to low according to the GRADE framework.

### 3.4. Robustness Analysis

A robustness analysis was conducted to compare the diagnostic performance of earlier (VivaScope^®^ 1000) and newer-generation (VivaScope^®^ 2500) EVCM devices for the diagnosis of SCC in tissue specimens. The pooled sensitivity did not differ significantly between generations (newer: 83.5%, 95% CI: 68.3–92.3; older: 95.5%, 95% CI: 55.2–99.7; *p* = 0.347), nor did the pooled specificity (newer: 95.5%, 95% CI: 90.7–97.9; older: 95.5%, 95% CI: 55.2–99.7; *p* = 0.992). For margin assessment, the available data were insufficient to perform a meaningful sensitivity analysis.

## 4. Discussion

While conventional histopathological examination of excised tissue remains the diagnostic gold standard, it is resource-intensive, time-consuming, and might delay decision-making [5,6]. EVCM, by enabling rapid visualization of SCC-specific morphological features, has emerged as a promising novel diagnostic tool [5,9]. We conducted a systematic review and meta-analysis to determine the diagnostic accuracy of EVCM for the diagnosis of SCC in skin tissue specimens and margin assessment in micrographic surgery.

We identified six eligible studies comprising a total of 288 tissue specimens, of which four studies evaluated the diagnostic accuracy of EVCM for SCC diagnosis in tissue specimens and two studies focused on margin assessment in micrographic surgery. For SCC diagnosis in tissue specimens, the pooled sensitivity and specificity were 85.1% and 95.5%, respectively, with moderate certainty of evidence. For margin assessment, pooled sensitivity and specificity were 89.9% and 96.1%, respectively, with low certainty of evidence. A trend toward improved sensitivity and specificity with newer devices was apparent, without statistical significance.

### 4.1. Comparison with Relevant Literature

Confocal microscopy has been studied in both in vivo and ex vivo settings since the late 20th century across a wide range of dermatologic conditions [25,26,27]. Multiple studies have demonstrated that EVCM is capable of depicting characteristic morphological features of SCC [9,28,29]. However, the majority of published work to date has been predominantly qualitative, focusing on technical optimization and the description of diagnostic criteria. In contrast, only a limited number of studies have reported quantitative diagnostic accuracy metrics, such as sensitivity and specificity. To the best of our knowledge, this is the first systematic review and meta-analysis to comprehensively evaluate the diagnostic accuracy of EVCM for SCC diagnosis in tissue specimens and margin assessment in micrographic surgery by synthesizing the available quantitative evidence.

### 4.2. Clinical Implications

EVCM holds the potential to complement and in certain cases to replace conventional histopathological examination. We differentiate between two distinct clinical contexts: SCC diagnosis in tissue specimens and surgical margin assessment in micrographic surgery. Given the differing clinical requirements and time constraints associated with these applications, we evaluated them separately.

SCC diagnosis, typically performed using punch or shave biopsy, generally occurs days to weeks before definitive surgical intervention and is therefore subject to fewer time constraints. In this setting, high sensitivity is essential to minimize missed diagnoses, while high specificity is required to avoid unnecessary surgical interventions. In our analysis, EVCM demonstrated moderate sensitivity and high specificity for SCC detection (pooled estimates of 85.1% and 95.5%, respectively). Although these results do not support replacement of conventional histopathology as the primary diagnostic standard, they suggest that EVCM may selectively be used as an adjunct to conventional histology in specific clinical scenarios. In particular, when rapid decision-making is desired, EVCM may serve as a confirmatory diagnostic tool, capitalizing on its high specificity to reliably rule in SCC and thereby facilitate timely surgical excision. The surgical excision specimen should still undergo conventional histopathological examination to ensure that the risk of misdiagnosis remains minimal.

Margin assessment represents a fundamentally different clinical scenario, typically encountered in Mohs or other micrographic surgical procedures, where rapid intraoperative feedback on whether a “positive margin”, defined as the presence of tumor at the resection margin, is present would be highly beneficial. For margin assessment, the pooled sensitivity and specificity of 89.9% and 96.1%, respectively, are promising. However, the certainty of evidence for these estimates is low, primarily due to the inclusion of only two studies and the small sample size of the analysis. To derive meaningful clinical implications for the use of EVCM in margin assessment, further large-scale studies are required. If these promising results are confirmed, EVCM could become a valuable diagnostic technique for reducing intraoperative waiting times during micrographic surgery.

### 4.3. Strengths and Limitations

To the best of our knowledge, this work provides the first extensive quantitative evaluation of the diagnostic accuracy of EVCM for the detection of SCC in tissue specimens and for margin assessment during micrographic surgery. The review was performed in line with established standards for diagnostic accuracy research, including prospective registration in PROSPERO, compliance with PRISMA guidelines, and the application of the QUADAS-2 and GRADE frameworks to evaluate risk of bias and certainty of evidence.

Several limitations should also be acknowledged. First, the number of eligible studies and the sample size available for quantitative synthesis were small, particularly for margin assessment. This limited the precision of the pooled estimates, precluded predefined subgroup analyses of micrographic surgery techniques for margin assessment, and prevented a formal assessment of publication bias for margin assessment. Together with concerns regarding risk of bias and applicability to our research question in some of the included studies, this led to downgrading the certainty of evidence to moderate for the diagnostic accuracy measures of SCC diagnosis in tissue specimens and to low for the diagnostic accuracy measures of margin assessment. Second, most included studies were single-center investigations with heterogeneous levels of reader expertise and highly specific study designs and specimen preparation protocols, which may introduce selection bias and limit the generalizability of our findings to other clinical settings. Third, the predefined subgroup analysis comparing the diagnostic accuracy of EVCM across different micrographic surgery techniques could not be performed due to insufficient data, and therefore heterogeneity could not be explored as planned.

### 4.4. Future Research

The findings of this systematic review and meta-analysis highlight the need for larger, prospective, multicenter studies using standardized protocols, particularly for margin assessment. The integration of artificial intelligence with digital EVCM imaging may further enhance diagnostic accuracy in the future [30,31]. Early data, primarily derived from studies on basal cell carcinoma (BCC), are promising and are expected to improve as larger, well-annotated EVCM image datasets become available for the training and validation of machine-learning models [30,32,33]. In general, EVCM has been more extensively studied in BCC. We are currently working on a systematic review and meta-analysis on the diagnostic accuracy of EVCM for BCC diagnosis and margin assessment, the results of which will allow direct comparison with the findings of the present study upon publication.

Apart from the diagnosis and margin assessment of SCC using EVCM, the detection of high-risk features (such as perineural invasion or desmoplasia) should be further investigated, as subsequent clinical management, including follow-up strategies, depends on their identification.

## 5. Conclusions

EVCM demonstrates moderate sensitivity and high specificity for the diagnosis of SCC in tissue specimens and may be used selectively as an adjunct to conventional histology, for example as a rapid confirmatory diagnostic tool capitalizing on its high specificity when expedited surgical planning is desired and conventional histopathological examination of the surgical excision specimen ensures a minimal risk of misdiagnosis.

For margin assessment in micrographic surgery of SCC, EVCM demonstrates promising diagnostic accuracy and has the potential to substantially improve surgical workflows by providing rapid intraoperative feedback. However, given the currently limited and low-certainty evidence, EVCM cannot yet be recommended for margin assessment in routine clinical practice.

## Figures and Tables

**Table 1:** Main Characteristics of Included Studies. EVCM = ex vivo confocal laser scanning microscopy.

Study	Patients, n	Specimens, n	EVCM Device	Diagnostic Accuracy Assessment
Gellrich et al., 2024 [21]	Not provided	11	VivaScope^®^ 2500 *	Margin assessment
Horn et al., 2007 [22]	20	20	VivaScope^®^ 1000 *	Diagnosis in tissue specimens
Messner et al. 2024 [7]	50	50	VivaScope^®^ 2500 *	Diagnosis in tissue specimens
Mu et al., 2016 [24]	Not provided	64	VivaScope^®^ 2500 *	Margin assessment
Ogrzewalla et al., 2022 [23]	23	23	VivaScope^®^ 2500 *	Diagnosis in tissue specimens
Vladimirova et al., 2022 [5]	91	120	VivaScope^®^ 2500 *	Diagnosis in tissue specimens

* Lucid Inc., Rochester, NY, USA.

## Data Availability

The datasets generated and analyzed during the current study are available from the corresponding author on reasonable request. Access to the data will be granted for legitimate academic purposes.

## Supplementary Materials

The following supporting information can be downloaded at: https://www.mdpi.com/article/10.3390/diagnostics16101539/s1, Table S1: Search Strategy for Searching PubMed and Embase via Ovid; Table S2: QUADAS-2 Assessment of Risk of Bias and Applicability Concerns Across Included Studies; Figure S1: Graphical Summary of QUADAS-2 Assessment; Figure S2: Funnel Plot of Individual Study Diagnostic Odds Ratios of EVCM for the Detection of SCC.

## Abbreviations

The following abbreviations are used in this manuscript:

SCC	Squamous cell carcinoma
EVCM	Ex vivo confocal laser microscopy
H&E	Hematoxylin-eosin
PRISMA	Preferred Reporting Items for Systematic Reviews and Meta-Analyses
PROSPERO	Prospective Register of Systematic Reviews
AI	Artificial intelligence
QUADAS	Quality Assessment of Diagnostic Accuracy Studies
GRADE	Grading of Recommendations Assessment, Development and Evaluation
CI	Confidence interval
AUC	Area under the summary receiver operating characteristic curve
BCC	Basal cell carcinoma
