# Supplementary material for: The Diagnostic Accuracy of Ex Vivo Confocal Laser Scanning Microscopy for Squamous Cell Carcinoma: A Systematic Review and Meta-Analysis
Source: Diagnostics (Basel). 2026 May 19;16(10):1539. doi: 10.3390/diagnostics16101539 (PMC13206270; doi:10.3390/diagnostics16101539)
Supplement: Supplementary file 1 [file diagnostics-16-01539-s001.zip › diagnostics-4171687-supplementary.pdf]

Supplementary Materials

Supplementary Tables

Supplementary Table S1

1	**Tumor**	(“squamous“ AND “cell“ AND “carcinom*”)<br>OR “SCC*”<br>OR “spinaloma*”<br>OR “epidermoid carcinom *”<br>OR “prickle cell carcinom *”<br>OR (“skin” AND (“tumor*” OR “tumour*”))<br>OR “MOH*”
2	**Diagnostic accuracy measures**	“sensitivit*”<br>OR “specificit*”<br>OR ((“positive” AND “predictive” AND “value*”) OR “PPV”)<br>OR ((“negative” AND “predictive” AND “value*”) OR “NPV”)<br>OR “accurac*”
3	**Imaging modalities**	(“confocal” AND “microscop*”)<br>OR “CLSM”<br>OR (“laser” AND (“scanning” OR “scan”) AND “microscop*”)<br>OR “LSCM”<br>OR “LSM”<br>OR “FCM”<br>OR (“fluorescence” AND “microscop*”)<br>OR ((“fluorescence” OR “reflectance”) AND “mode”)<br>OR “RCM”<br>OR ((“confocal” OR (“ex” AND “vivo”)) AND “imag*”)<br>OR (“ex” AND “vivo” AND “histology”)<br>OR “EVCM”<br>OR (“optical” AND “sectioning” AND “microscop*”)<br>OR (“digital” AND “microscop*”)<br>OR “OCT”<br>OR (“optical” AND “coherence” AND “tomography”)<br>OR (((“optical” AND “coherence”) OR “coherent”) AND “imag*”)<br>OR “LC-OCT”<br>OR (((“line” AND “field”) OR “line-field”) AND “confocal” AND “optical” AND “coherence” AND “tomography”)<br>OR (“multimodal” AND “imag*”)
4	**Final search**	1 AND 2 AND 3

Supplementary Table S1. Seach Strategy for Searching PubMed and Embase via Ovid. SCC = squamous cell carcinoma; MOH = Micrographic (Mohs) Surgery; PPV = positive predictive value; NPV = negative predictive value; CLSM = confocal laser scanning microscopy; LSCM = laser scanning confocal microscopy; LSM = laser scanning microscopy; FCM = fluorescence confocal

microscopy; RCM = reflectance confocal microscopy; EVCM = ex vivo confocal microscopy; OCT = optical coherence tomography; LC-OCT = line-field optical coherence tomography.

Supplementary Table S2

STUDY	RISK OF BIAS*				APPLICABILITY CONCERNS*		
	Patient Selection	Index Test	Reference Standard	Flow and Timing	Patient Selection	Index Test	Reference Standard
Gellrich et al., 2024	green	green	blue	green	red	green	green
Horn et al., 2006	red	green	green	green	red	green	green
Messner et al., 2024	red	green	green	green	green	green	green
Mu et al., 2016	red	green	blue	red	red	green	green
Ogrzewalla et al., 2022	green	green	blue	green	green	green	green
Vladimirova et al., 2022	blue	green	green	green	green	green	green

Supplementary Table 2. QUADAS-2 Assessment of Risk of Bias and Applicability Concerns Across Included Studies. QUADAS-2 = Quality Assessment of Diagnostic Accuracy Studies 2 tool.

* Green dots indicate low risk, red dots indicate high risk, and blue dots indicate unclear risk.

Supplementary Figures

Supplementary Figure S1

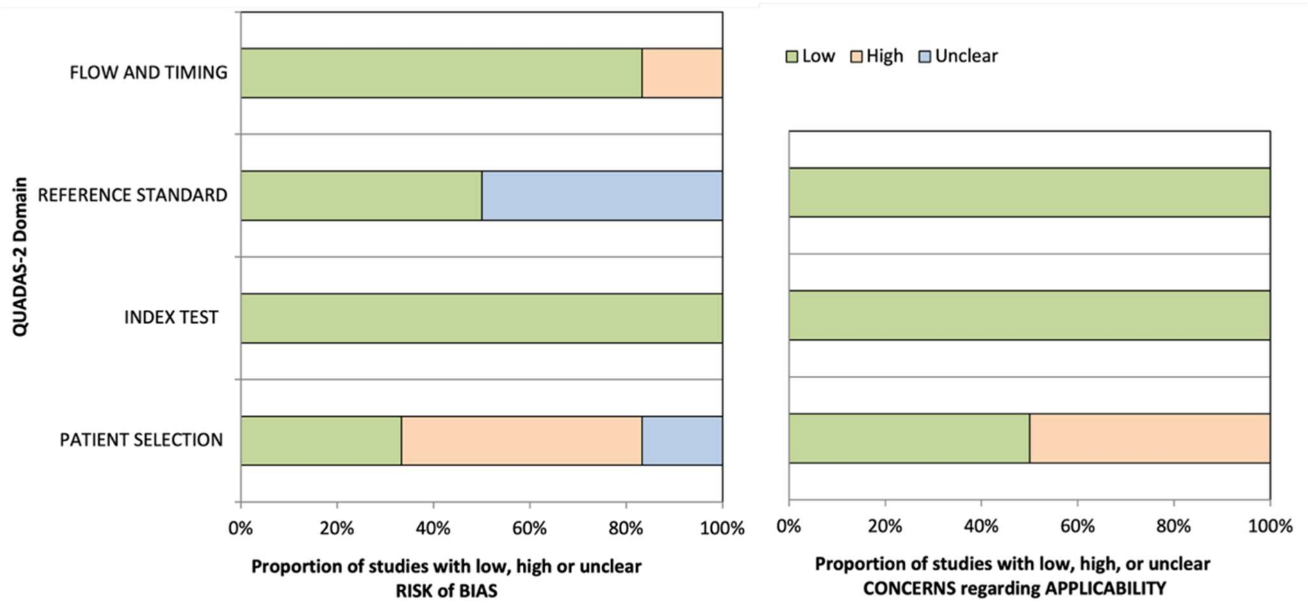

Supplementary Figure S1. Graphical Summary of QUADAS-2 Assessment. QUADAS-2 = Quality Assessment of Diagnostic Accuracy Studies 2 tool.

Supplementary Figure S2

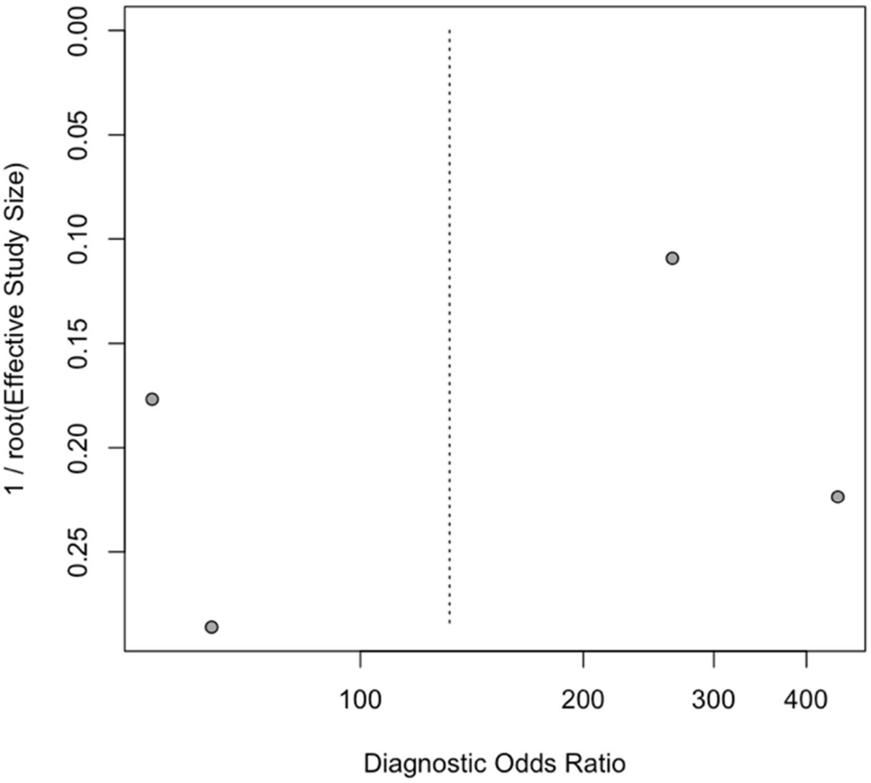

Supplementary Figure S2. Funnel Plot of Individual Study Diagnostic Odds Ratios of EVCM for the Detection of SCC. Each point represents an individual study's diagnostic odds ratio plotted against its standard error. The vertical line indicates the pooled effect estimate from the random-effects model. Visual inspection allows assessment of potential small-study effects or publication bias. EVCM = ex vivo confocal microscopy; SCC = squamous cell carcinoma; CI = confidence interval.
